# Development and Validation of the Augsburg Nasopharyngeal Applicator: Enhancing Efficacy in Nasal Route Brachytherapy

**DOI:** 10.1016/j.adro.2025.101896

**Published:** 2025-08-30

**Authors:** Jerome Jean-Joseph, Christoph Westerhausen, Johannes Doescher, Bruno Maerkl, Zoha Roushan, Maria Neu, Tilman Janzen, Klaus-Henning Kahl, Georg Stueben, Nikolaos Balagiannis

**Affiliations:** a Medical Physics Department, Augsburg University Hospital, Augsburg, Germany; b Physiology, Institute of Theoretical Medicine, University of Augsburg, Augsburg, Germany; c Department of Otorhinolaryngology, Augsburg University Hospital, Augsburg, Germany; d Department of Pathology and Molecular Diagnostics, Augsburg University Hospital, Augsburg, Germany; e Radiotherapy Department, Faculty of Medicine, University of Augsburg, Augsburg, Germany; f Comprehensive Cancer Center Augsburg, Faculty of Medicine, University of Augsburg, Augsburg, Germany; g Comprehensive Cancer Center Alliance WERA, Augsburg, Germany; h Bavarian Cancer Research Center, Augsburg, Germany

## Abstract

**Purpose:**

This study evaluates the Augsburg Nasopharyngeal Applicator (ANA), a novel nasal brachytherapy device designed for early-stage nasopharyngeal carcinoma (T1–T2 stages). ANA leverages nasal anatomy to overcome limitations of oral applicators, optimizing tumor targeting while sparing adjacent tissues, such as the soft palate and oral mucosa.

**Methods and Materials:**

ANA was developed using sagittal computed tomography-based anatomic measurements and computer-aided design modeling. Structural integrity was validated through nonlinear finite-element analysis, mechanical stress testing (including Euler buckling tests), and displacement testing (30 min vibration at 5 Hz with 2 cm amplitude). Dosimetry was verified using radiochromic film with 3%/3 mm gamma analysis criteria, following the TG-43 formalism for dose calculation. Insertion feasibility was assessed in a postmortem model under institutional autopsy protocols.

**Results:**

ANA (with nylon 6/6 catheter) withstood displacements up to 30 mm without failure. Simulated motion tests demonstrated positional stability (<1 mm displacement). Dosimetry achieved a 97.5% gamma pass rate (clinical acceptability threshold: 95%), with the 20 mm curvature configuration reducing soft palate doses by >50% compared to standard oral applicators (eg, Rotterdam design). Postmortem insertion was completed in 10 min, with endoscopic confirmation of positioning accuracy within 1 mm.

**Conclusions:**

ANA demonstrates precise positioning, mechanical stability under simulated physiologic motion (<1 mm displacement), and clinically significant dose sparing (>50% reduction to the soft palate with the 20 mm curvature configuration). Its nasal approach and anatomic adaptability position it as a promising alternative to oral applicators. These proof-of-concept findings support the need for phase 1/2 clinical trials to evaluate safety and efficacy in patients.

## Introduction

The nasopharyngeal carcinoma exhibits a bimodal age distribution (peaks: 15–24 and 65–79 years) with a global incidence of 2.12/100,000.^1^ Brachytherapy is indicated for early-stage (T1–T2, N0–N1), post-external beam radiation therapy residual, or inoperable recurrent nasopharyngeal carcinoma.^2^, ^3^, ^4^, ^5^ However, conventional oral applicators (eg, Rotterdam) face critical limitations: procedural discomfort from gag reflexes, suboptimal positioning accuracy, and inadequate sparing of adjacent organs at risk (OARs) like the soft palate.^6^^,^^7^

To overcome these constraints, we developed Augsburg Nasopharyngeal Applicator (ANA)—a nasally inserted, anatomically adaptive device exploiting the nasal passage’s linear alignment with the nasopharynx. ANA’s customizable curvature aims to:

(1) Enhance patient tolerability by avoiding gag reflexes.
(2) Improve tumor targeting through computed tomography (CT)-guided positioning.
(3) Reduce OAR doses via optimized geometric configuration.

This study evaluates the technical feasibility of the ANA as a precision-guided alternative with potential for clinical translation.

## Methods and Materials

### System overview

#### Catheter

The catheter is fabricated from flexible, biocompatible nylon 6/6 tubing (French size 6, Zeus) with a Shore hardness of D85 and certified to United States pharmacopeia Class VI standards. To ensure safety and mechanical performance during use, a visual marker is integrated along the shaft to indicate the maximum allowable bending curvature. This marker serves as a guide for clinicians during insertion, helping to prevent overstressing the material and ensuring consistent, reproducible shaping of the catheter.

The catheter’s radiation stability was evaluated by delivering a dose of 200 Gy at a depth of 1 cm in a Plexiglas phantom. Postirradiation analysis showed no measurable degradation in mechanical performance: the catheter maintained its flexibility, structural integrity, surface properties, hardness, and tensile strength. Additionally, no discoloration was observed, suggesting excellent resistance to radiation-induced oxidation or crosslinking at the applied dose.

#### Stopper

A 3 cm segment of medical-grade polyvinyl chloride (PVC) tubing (French 8) serves as the stopper, acting as a flexible connector between the catheter and the corpus, and as a tip for the open-ended catheter. This glue-free design provides several advantages: the stopper’s elasticity distributes stress evenly, reducing the risk of localized failure, and offers controlled flexibility to accommodate anatomic variations, thereby ensuring smoother contact. The absence of adhesive improves device reliability and longevity by eliminating the risk of glue failure. Additionally, the soft, glue-free tip minimizes the risk of irritation or injury to the nasopharynx during insertion and use.

#### Corpus

The core structure, or corpus, is made from transparent, medical-grade PVC (French 16, Shore hardness A70). It houses both the catheter and the mandrel, providing essential structural support. An adjustable perforation system allows for precise size modifications, enabling the applicator to conform to individual patient anatomy within the nasopharynx.

#### Loop

The loop consists of a polyamide mandrel (French 3, Zeus) shaped to apply controlled force to the corpus for precise bending and orientation. This component allows for fine adjustment of the applicator’s tilt (up to 22°), enabling targeted lateral dose delivery to specific areas within the nasal cavity. The loop ensures secure positioning and maintains a stable connection between the applicator and the vault of the nasopharynx.

Additionally, a montage video (ANA-montage.mp4) demonstrating the assembly of the device is provided as supplementary material.

**Figure 1:**
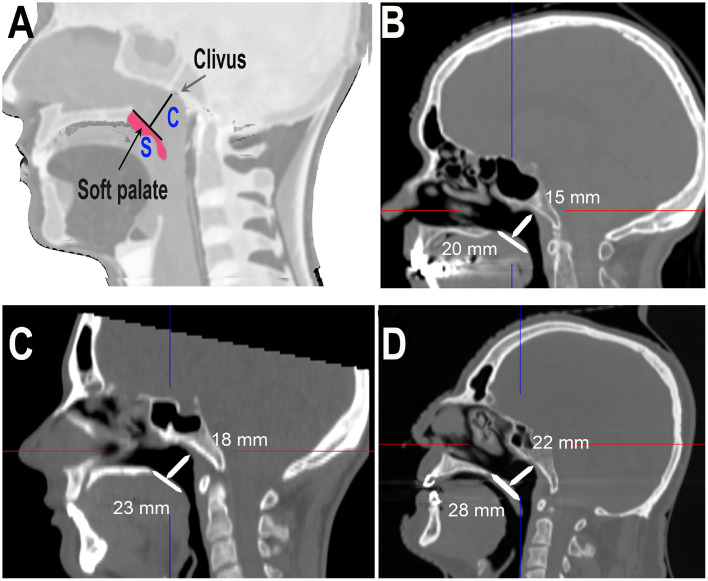
(A) S: the section of the soft palate excluding the uvula; C: the distance from the midpoint of S to the clivus. (B) A 24-year-old female, 158 cm, 46 kg. (C) A 49-year-old male, 175 cm, 73 kg. (D) A 71-year-old male 190 cm, 107 kg.

Our approach to designing ANA is based on 2 key measurements^8^ obtained through sagittal CT imaging (Fig. 1A).

These measurements (S&C) are pivotal in determining ANA design and placement strategy: The linear segment of the soft palate (S) serves as a critical positioning reference for our applicator. We have identified the terminus of this straight portion as the optimal location for ANA tip. This anatomic landmark is strategically chosen because it precedes the uvula, which guides the bending of ANA tip.

By using this consistent anatomic feature, we can achieve reproducible applicator placement after each therapy fraction. Additionally, the distance to the clivus (C) provides important context for ensuring proper curvature and positioning of the device. “S” guides the initial positioning, while “C” helps fine-tune the applicator's curvature and final placement. This dual-measurement approach enhances the precision and adaptability of our technique across various patient profiles (Fig. 1B-D).^9^

#### Design and finite-element analysis

We conducted a nonlinear finite-element analysis (FEA) using SOLIDWORKS (version 2023, Dassault Systèmes) to numerically solve the differential equations governing catheter bending under deformations.^10^

A detailed description of the mathematical modeling of the bending mechanism is provided in Annex A, while the FEA setup is outlined in Annex B. This approach discretizes the intricate catheter geometry into manageable elements, enabling comprehensive analysis of its behavior under applied forces.

The FEA model confirms catheter safety at displacements ≤30 mm, directly ensuring clinical durability during insertion.

#### ANA phantom design and validation

**Figure 2:**
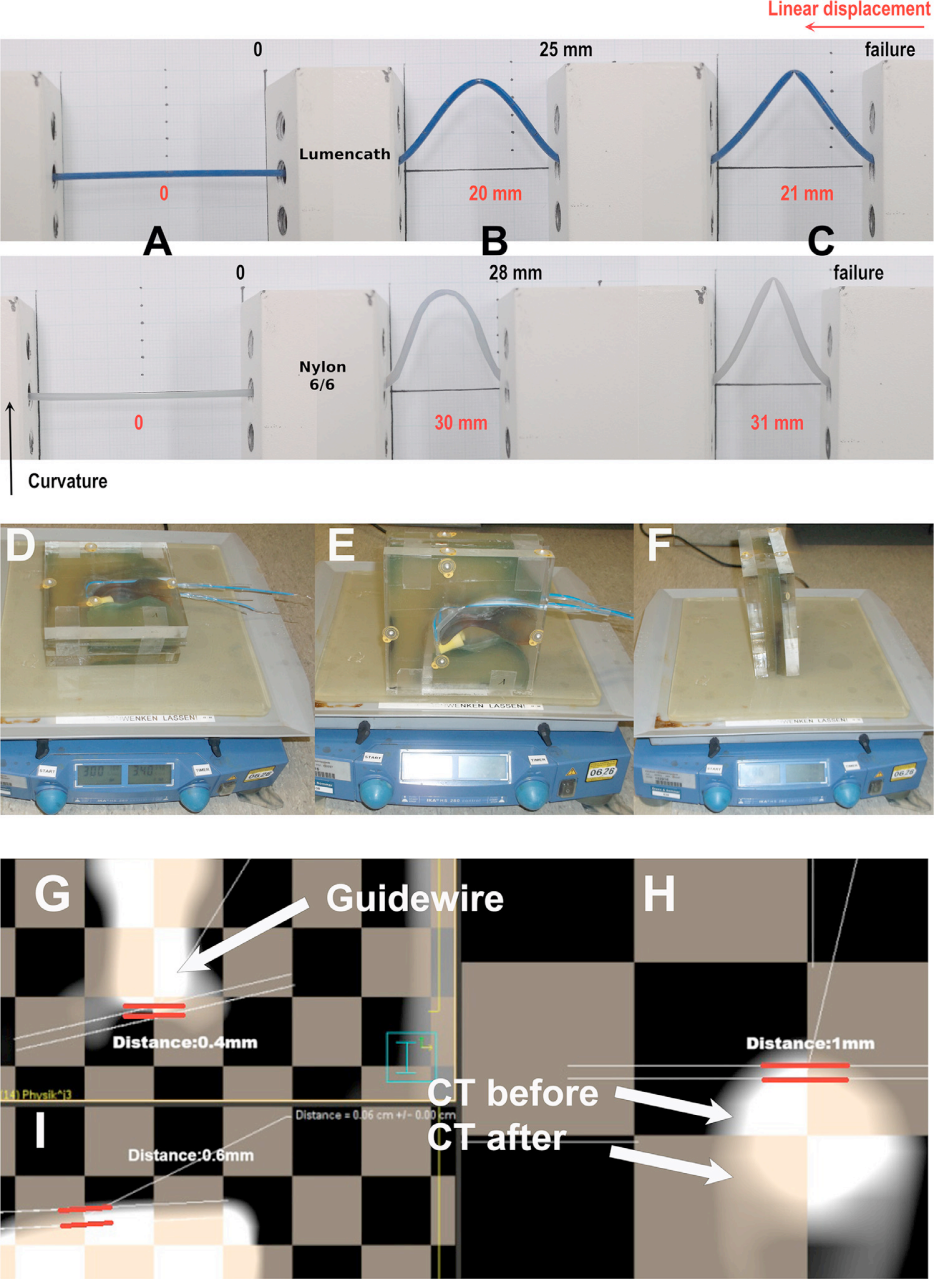
Mechanical and vibration testing of ANA with CT comparisons. *Abbreviations:* ANA = Augsburg Nasopharyngeal Applicator; CT = computed tomography.

We developed a custom-designed phantom specifically for this study (Fig. 2D-F) to replicate relevant nasopharyngeal anatomy and tissue characteristics. Its dimensions were based on axial CT slices from a 49-year-old patient, ensuring anatomic realism for accurate applicator testing. The phantom featured a layered construction: two 2 cm sheets of water-equivalent polymethyl methacrylate on either side, a central 2.5 cm tissue-equivalent bolus layer, and a radiochromic film embedded at mid-depth for dose measurement.

#### Bending and stability constraints

To assess the buckling resistance of the catheter, we developed a custom Euler Buckling Test setup (Fig. 2A-C).^11^ Our modified configuration consists of 2 lead blocks, each measuring 2.5 cm × 14 cm × 5 cm, positioned at adjustable distances to simulate pinned-pinned boundary conditions. The catheter specimen is securely fixed at both ends, ensuring axial alignment before applying compressive displacement. As the linear displacement increases, the catheter undergoes elastic buckling, forming characteristic curvature patterns until reaching the critical buckling load, after which structural failure occurs. This setup allows for direct observation of the buckling mode and quantification of maximum curvature and failure displacement, providing insights into the catheter’s structural stability under axial compression.

(A–C) Sequential stress test illustrating the progression from rest (A), through maximum curvature (B), to failure (C) for 2 catheter types: Lumencath (blue, 6F, polyamide, Elekta, Sweden) and nylon 6/6 (natural translucent, 6F, Zeus). (D–F) ANA positioning in the vibration apparatus for transversal (D), longitudinal (E), and lateral (F) orientations. (G–I) CT images comparing pre- and postvibration ANA alignment and displacement in lateral (G), transversal (H), and longitudinal (I) planes.

This process continues until the catheter begins to exhibit kinking behavior, which signifies the limit of its maximum safe bending radius and flexural strength. Beyond this threshold, the catheter cannot maintain its structural integrity, transitioning from elastic deformation to permanent kinking. This critical point defines the maximum allowable curvature *κ*_max_ as quantified by the minimal acceptable bending radius *R*_min._

The test results provide essential data for determining the catheter’s mechanical limits, ensuring that it can withstand the stresses encountered during clinical use without failure.

Observations indicate that the nylon 6/6 catheter can bend to a curvature of approximately 28 mm over a linear displacement of 30 mm, while the Lumencath exhibits a curvature of 25 mm over a displacement of 20 mm.

The materials exhibit distinct mechanical properties—specifically, stiffness, and flexibility. These discrepancies arise from the difference in shore D hardness, with the Lumencath being more rigid in this case.

This comparative analysis suggests the nylon 6/6 catheter achieves a more gradual bend profile under equivalent displacement conditions. The test ensures the catheter withstands bending stresses up to 30 mm displacement without kinking.

As ANA is intended for single-use intranasal application, repeated flexural cycles are not anticipated in clinical practice. Therefore, the bending and buckling tests conducted in this study are designed to simulate the maximum expected deformation during a single insertion and positioning session. This approach ensures structural integrity under the most demanding one-time clinical use scenarios. Repeated fatigue testing was deemed unnecessary for this single-use application.

To evaluate the positional stability of the ANA, a displacement test was performed using a laboratory shaker (HS 260 control, IKA-Werke GmbH & Co. KG) (Fig. 2D-F). The setup included a tissue-equivalent phantom with the ANA in situ, positioned on the shaker platform. The shaker was programmed with a 2 cm amplitude and 5 Hz frequency to simulate typical patient movements,^12^ such as breathing, head tossing, head shaking, and speaking.

The experiment was performed over a total duration of 90 minutes, with 30 minutes of agitation along each of the 3 principal axes (transversal, longitudinal and lateral). The stability of the applicator was assessed by acquiring high-resolution CT (Somatom, Siemens) scans (1 mm slices) of the phantom before and after the test.

Results from our treatment planning system (Pinnacle 16.2, Philips Radiation Oncology) demonstrated minimal displacement of ANA, with measured positional shifts equal to or less than 1 mm along all axes (Fig. 2G-I).

A video (ANA-vibration.mp4) recording of the test is provided as supplementary material.

#### Applicator commissioning

**Figure 3:**
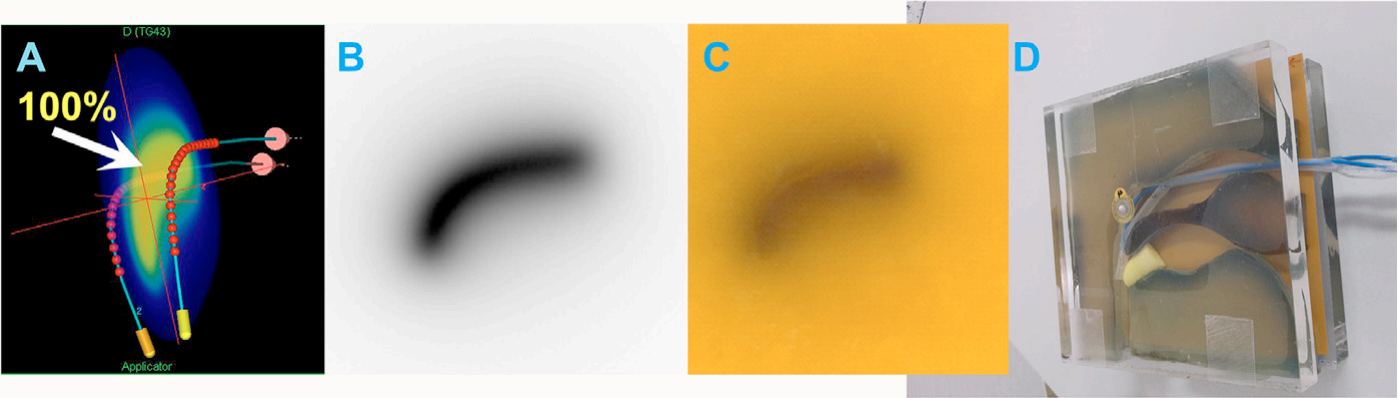
(A) Isodose map. (B) DICOM RT dose. (C) Radiochromic film. (D) ANA in Phantom with radiochromic film. *Abbreviations:* ANA = Augsburg Nasopharyngeal Applicator; RT = radiation therapy.

To optimize the applicator’s performance, we conducted initial validation using radiochromic film (EBT3, Gafchromic, Ashland Inc) (Fig. 3C, D).^13^ This step was crucial in determining the precise offset and appropriate indexer length for our high-dose rate afterloader (MicroSelectron, Elekta AB), which used an iridium-192 (Ir-192) source (initial activity: 370 GBq; source dimensions: 3.5 mm length × 0.6 mm diameter).^14^ Dwell positions were optimized for target homogeneity along the active catheter length. Dose calculations were performed according to the TG-43 formalism.

#### Irradiation setup

The digitized films were analyzed to extract dose information, which was then compared with the calculated dose distributions (Fig. 3B). The combination of calculated isodose maps, DICOM radiation therapy doses, and high-resolution film dosimetry provided a comprehensive view of the dose distribution.^15^^,^^16^

#### Postmortem model

ANA was evaluated during a postmortem examination^17^ conducted for clinical purposes (Autopsy case 25/12: 67-year-old male, non-nasopharyngeal etiology confirmed as cardiovascular failure). In alignment with institutional autopsy guidelines and local legal statutes governing postmortem procedures, ANA testing was performed under the condition that it posed no additional risk of harm, preserved anatomic integrity, and did not interfere with diagnostic objectives.

ANA, designed for noninvasive tissue compatibility assessment, was tested postmortem using methods indistinguishable from standard autopsy practice. Postprocedure verification confirmed that no anatomic structures were altered or compromised. All findings related to the primary cause of death remained unaffected, and no personal identifiers were retained in device testing documentation.

Single-cadaver validation establishes proof of concept; multisubject trials are planned for clinical translation.

#### Preparation

**Figure 4:**
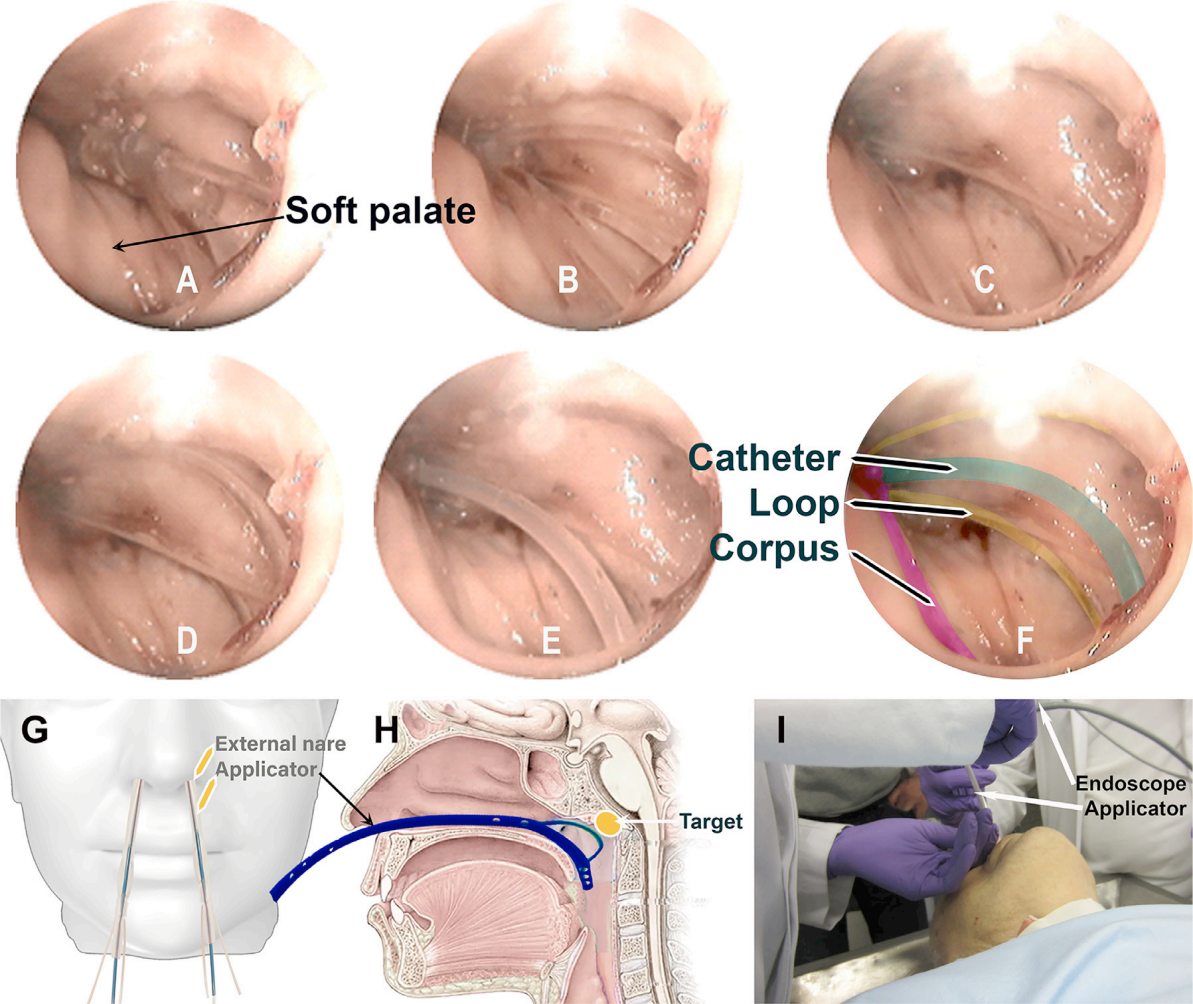
Steps of ANA insertion: (A) initial placement of device near the soft palate/uvula, (B) positioning of ANA, (C) deployment of internal component, (D) device locked into position, (E) bending the catheter, and (F) final secured position of ANA. (G) Frontal view of nasopharynx applicator placement. (H) Sagittal view of ANA in position. (I) Endoscopic setup of ANA in postmortem trial. *Abbreviation:* ANA = Augsburg Nasopharyngeal Applicator.

The deceased individual was positioned supine on the examination table (Fig. 4I), with the head tilted back to optimize access to the nasal passages. This positioning provided a clear and unobstructed pathway for the instruments to reach the nasopharyngeal cavity. Standard precautions were taken to ensure that the head remained stable throughout the procedure, facilitating precise manipulation of the endoscopic and applicator instruments.

The endoscope (no 11101, Karl Storz)^18^ was gently inserted through the right nostril, providing a direct and clear view of the nasopharyngeal region. In postmortem conditions, real-time endoscopic visualization was used to guide the placement of the first applicator, which was introduced through the left nostril. It was secured in place using the loop, and the catheter was precisely bent to align with the roof and posterior wall of the nasopharynx. After removing the endoscope from the right nostril, the second applicator was inserted at the same level as the first, replicating the loop and catheter curvature.

In a clinical setting, CT imaging is mandatory for treatment planning and postinsertion validation of ANA to ensure precise positioning and optimal dose delivery. CT enables accurate delineation of the target volume and OAR, particularly when a second applicator is inserted in the contralateral nostril, where endoscopic visualization is not feasible because of anatomic constraints. In contrast, during the cadaver study, an endoscope was used solely to film the insertion process for documentation.

A video recording of the ANA insertion procedure (ANA-insertion.mp4) is provided as supplementary material.

## Results

**Figure 5:**
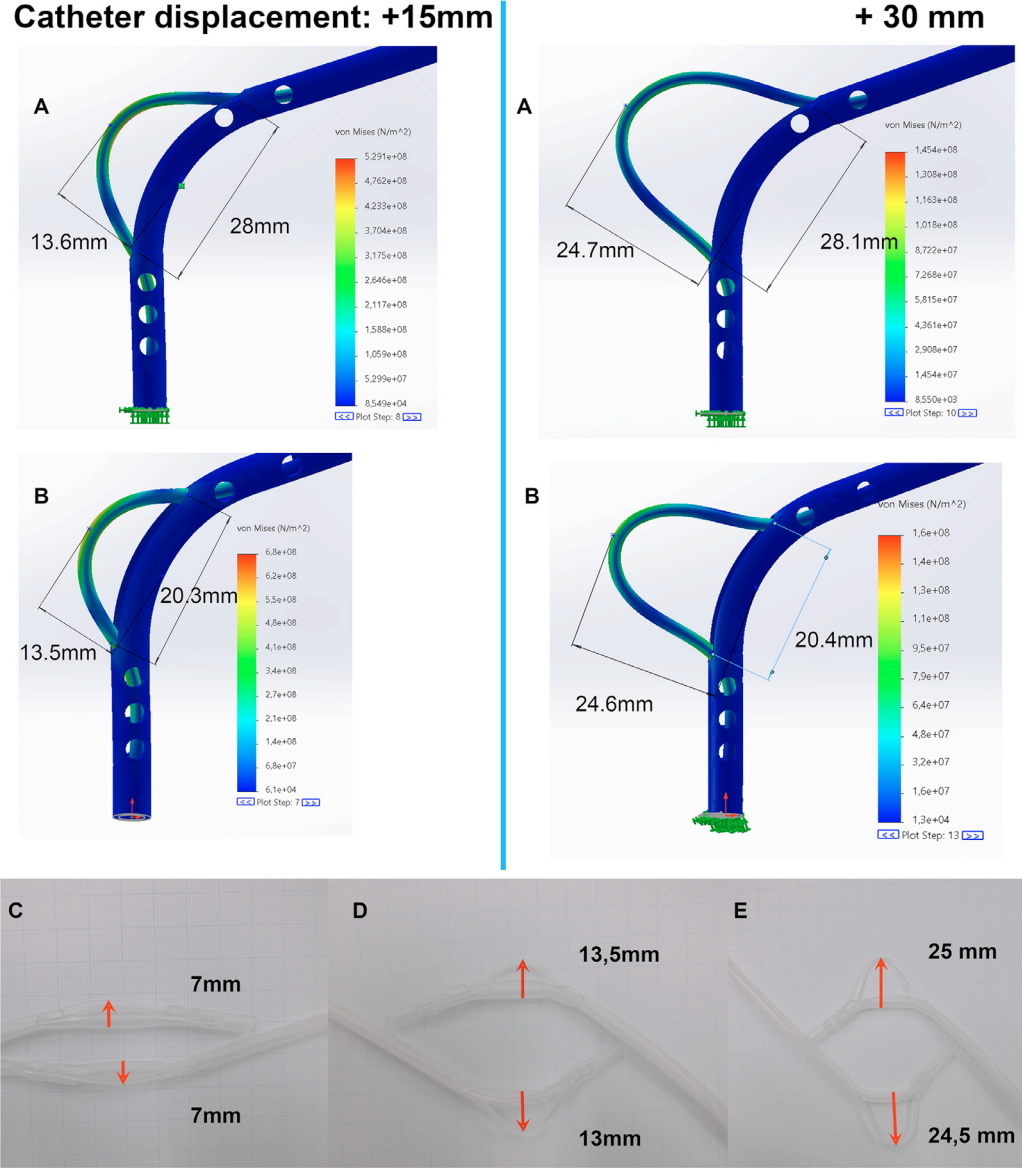
Von Mises stress distribution in ANA during simulated bending for both standard (A) and compact (B) configurations, shown under nylon 6/6 catheter displacements of +15 mm (left) and +30 mm (right) and comparing (C, D, E) compact (below) and standard (above) configurations at varying levels of catheter advancement.

In both configurations shown in Figure 5A, B, stress is primarily concentrated in the bending regions of the catheter. The highest levels are localized along the inner curvature, where compressive forces dominate (red zones), while the outer curvature, subjected to tensile forces, exhibits slightly lower stress levels. The blue zones indicate areas of minimal stress, illustrating the catheter's ability to effectively distribute mechanical loads along its length.

The surrounding corpus structure, which houses the catheter, plays a key role in absorbing the mechanical energy generated during deformation. Its flexibility allows it to accommodate catheter bending, thereby reducing stress on the catheter material and mitigating the risk of mechanical failure. This effect is particularly notable in the 30 mm displacement scenario: despite a pronounced bend of approximately 25 mm, the stress levels remain within the safe operational limits of nylon 6/6.

The nonlinear finite-element simulation provides valuable insights into the mechanical interaction between the catheter and its supportive corpus. The results confirm the system’s ability to withstand repeated bending cycles without structural compromise—demonstrating the device’s suitability for brachytherapy applications, where flexibility is critical.

Figure 5C-E illustrates the progressive bending behavior of the ANA in 3 configurations:

Configuration C (Fig. 5C) shows the ANA in a relaxed state, with a broad and gradual curvature. The loop at the tip is minimally engaged—representing conditions typically encountered during initial insertion along straight or mildly curved anatomic paths.

Configuration D (Fig. 5D) depicts an intermediate bend. The loop at the tip is more actively engaged, resulting in a tighter curvature suitable for navigating around anatomic obstacles, such as the nasal cavity.

Configuration E (Fig. 5E) demonstrates the catheter under maximum bending. This configuration may be required when redirecting the catheter away from the soft palate toward the region of the pharyngeal tonsils. Von Mises stress analysis confirms that even under these extreme conditions, the applicator maintains structural integrity.

### Dosimetric accuracy

**Figure 6:**
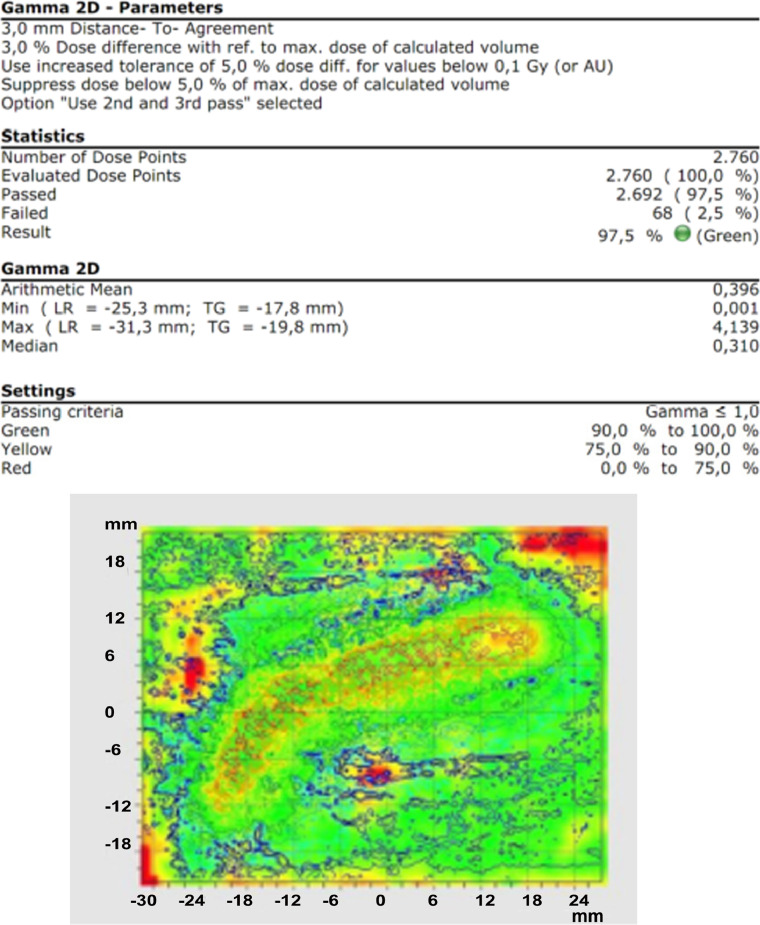
2D gamma analysis: comparison of irradiated vs calculated dose distribution.

The 2D gamma analysis (Fig. 6) was performed using the VeriSoft software package (PTW, Freiburg GmbH) with criteria of 3-mm distance-to-agreement and 3% dose difference. To account for uncertainties, regions failing the 3 mm or 3% criteria are highlighted in red. The overall gamma pass rate of 97.5% demonstrates excellent concordance, exceeding the 95% threshold recommended for clinical acceptability. Notably, most discrepancies were confined to low-dose-gradient regions at the periphery of the dose distribution. Dosimetric discrepancies arose primarily from film alignment, not dwell positioning, minimizing clinical impact.^19^

### Postmortem evaluation

ANA was successfully inserted (Fig. 4A-F) and subsequently removed without any complications. Through endoscopic visualization, it was confirmed that ANA achieved precise positioning within the nasopharynx, with the catheter securely aligned along the roof and posterior wall of the cavity. The loop maintained consistent and stable contact with the mucosal surface, ensuring the correct curvature of the catheter while preventing any obstruction or misalignment during the procedure. Both applicators were symmetrically positioned, accurately replicating the intended trajectory for optimal treatment delivery.

The entire procedure was completed in approximately 10 minutes. Once inserted, ANA remained stable throughout the duration of therapy without requiring manual support. For additional stability, fixation with a medical adhesive bandage is recommended.

### Comparison with Rotterdam Applicator configuration

**Figure 7:**
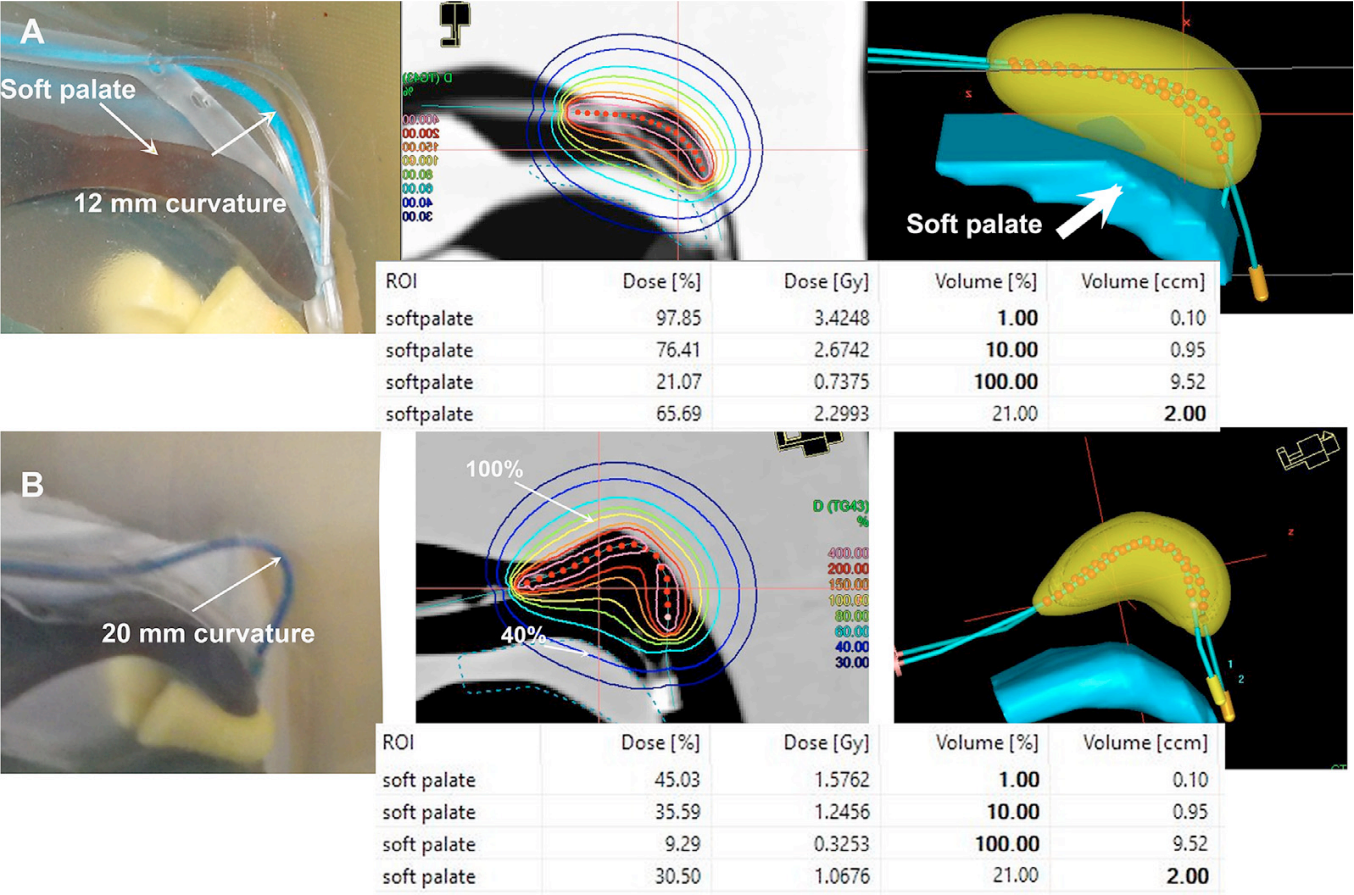
(A) Configuration standard applicator with 12 mm curvature. (B) 20 mm curvature (phantom, isodose, 3d dose, dose volume histogram).

ANA (Fig. 7) was configured with a 12 mm curvature to emulate the geometric profile of the Rotterdam nasopharyngeal applicator, enabling a standardized comparative framework. A prescribed dose of 3.5 Gy at 5 mm depth was delivered to the pseudopharyngeal tonsils, aiming for 100% target coverage while evaluating dose distribution to adjacent OAR, notably the pseudosoft palate.

Dosimetric analysis revealed significant differences in OAR sparing between the 12 mm and 20 mm curvature configurations. For the 12 mm curvature, the pseudosoft palate received a maximum dose of 3.4 Gy (97.8% of the prescribed dose) confined to 0.10 cm³ (1% of the structure), indicating a steep dose gradient. At larger volumes, the dose decreased to 2.7 Gy (76.4%) for 10% volume (0.95 cm³) and 0.7 Gy (21.1%) for the entire structure (9.5 cm³). Notably, the 2 cm³ volume (21% of the structure), a clinically critical region, received 2.3 Gy (65.7%).

In contrast, the 20 mm curvature configuration demonstrated superior sparing efficacy. The maximum dose to the pseudosoft palate was reduced to 1.6 Gy (45%), with progressive reductions across all volumes: 1.2 Gy (35.6%) at 10% volume, 0.3 Gy (9.3%) at 100% volume, and 1.1 Gy (30.5%) within the 2 cm³ region. This represents a 54% to 56% relative dose reduction in high-dose regions (1%-10% volumes) and a >50% reduction in the 2 cm³ volume compared to the 12 mm configuration.

## Discussion

The nasal approach to brachytherapy, as enabled by ANA, offers several significant advantages over traditional oral insertion techniques. Anatomically, the nasal passage provides a more direct and linear route to the nasopharynx, facilitating smoother insertion and more accurate positioning. ANA’s nasal route may reduce discomfort by avoiding gag reflexes (though clinical validation is pending)—a frequent complication associated with oral applicators—thereby reducing procedural risks that could compromise applicator placement accuracy.^20^

**Figure 8:**
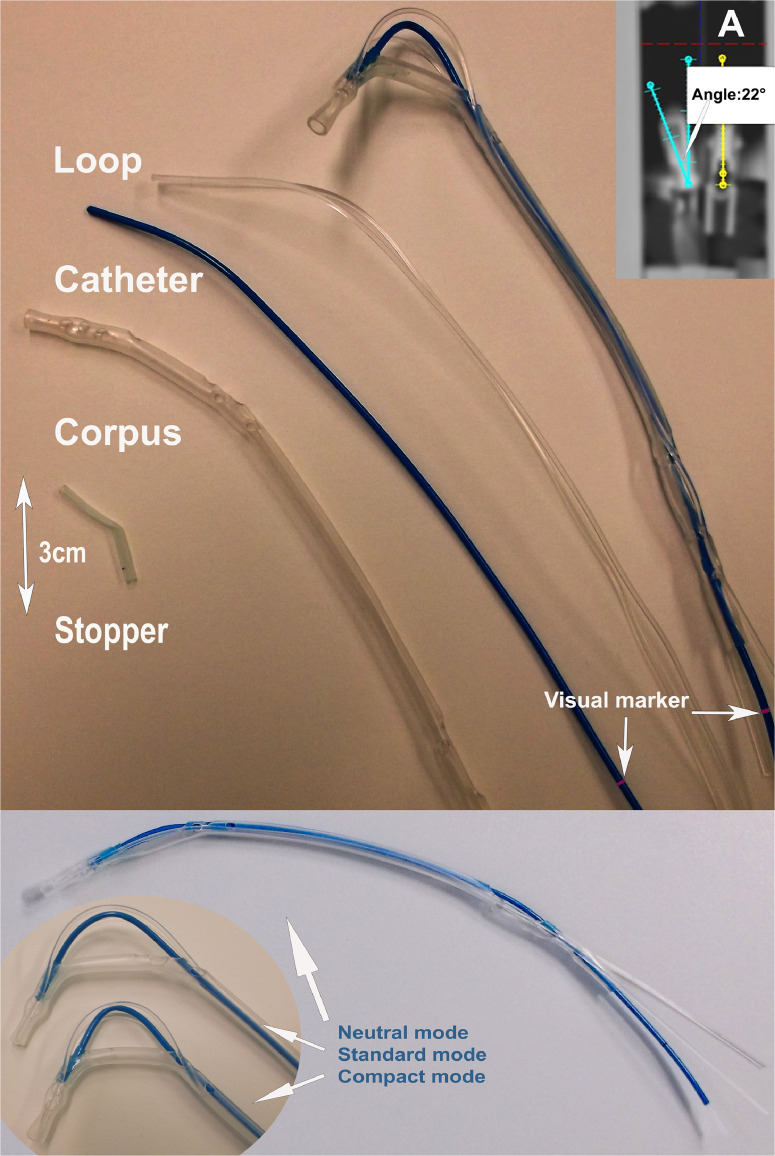
Components and configurations of ANA, including (A) CT view showing a tilted position. *Abbreviations:* ANA = Augsburg Nasopharyngeal Applicator; CT = computed tomography.

To accommodate interindividual variability in nasopharyngeal anatomy, ANA features an innovative, adjustable design with 2 distinct size configurations, compact and standard (Fig. 8). This adaptability allows clinicians to tailor the applicator to a broad range of patient anatomies without the need for multiple prefabricated models, streamlining inventory and improving workflow efficiency in clinical practice.

An additional clinical benefit of the nasal route lies in its potential to reduce infection risk. The nasal cavity generally harbors a lower microbial load compared to the oral cavity, which may translate into a reduced incidence of postprocedural infections. This is particularly relevant in immunocompromised patients or settings where strict infection control is essential.^21^ The associated reduction in prophylactic antibiotic use may further simplify posttreatment management and lower overall health care costs.^22^

In line with current trends toward personalized oncology, ANA also supports a patient-specific treatment paradigm through the integration of 3D printing. Customized applicators can be fabricated based on 2 reproducible anatomic measurements (Fig. 1A): (S) the linear length of the soft palate region and (C) the perpendicular distance from the posterior nasal spine to the clivus. These parameters inform a mathematical model that generates a digital design file, optimized for additive manufacturing via stereolithography.^23^

This individualized manufacturing workflow is designed to comply with the EU Medical Device Regulation ((EU) 2017/745, Article 2(3), Annex XIII) for custom-made Class IIa medical devices.^24^ The use of a nylon 6/6 catheter (D85 Shore hardness) provides mechanical flexibility, as demonstrated by FEA and buckling resistance testing under controlled laboratory conditions.

Repeated bending fatigue tests were not performed, as ANA is a sterile, single-use medical device intended for one treatment fraction. In clinical practice, mechanical stress is limited to initial insertion and minor positional adjustments. The bending and buckling tests included in this study were specifically designed to simulate the maximum mechanical deformation expected during such a single-use session. As such, they provide sufficient assurance of structural safety within the intended clinical context. Nonetheless, cyclic fatigue testing may be considered in future studies for regulatory support or comparison with reusable systems.

While these initial feasibility results demonstrate the technical viability of the design concept, comprehensive validation including biocompatibility assessment, clinical performance evaluation, and multisubject insertion studies will be required before clinical implementation. The current findings support the rationale for advancing to more extensive preclinical testing and eventual clinical trials to fully establish the device’s safety and efficacy profile.

Despite its promise, the nasal route is not without limitations. Potential complications include mucosal irritation and epistaxis, particularly in patients with narrow or fragile nasal passages.^25^ As such, careful patient selection and thorough operator training will be essential to ensure safe clinical implementation.

Future studies will assess in vivo stability and device behavior under physiologic conditions. Limitations of the present study include single-cadaver testing and preliminary comfort assessments.

## Conclusion

ANA represents a novel approach to nasopharyngeal brachytherapy that demonstrates preliminary technical feasibility through FEA, dosimetric validation, and postmortem evaluation. The nasal insertion route has the potential to offer theoretical advantages in patient tolerability and procedural workflow compared to conventional oral applicators, while the adjustable design may enable better conformance to individual patient anatomy.

This proof-of-concept study establishes the foundational engineering and dosimetric characteristics of the ANA design. However, clinical benefits regarding patient comfort, positioning accuracy, and infection control remain to be validated through systematic clinical investigation. The 3D-printable, patient-specific design concept aligns with emerging trends in personalized medical device manufacturing.

Given ANA’s single-use design, the current mechanical assessments—focused on maximum one-time deformation—are appropriate to ensure clinical safety during insertion and treatment. These preliminary findings provide a strong rationale for advancing toward more extensive preclinical validation and clinical trials.

## Disclosures

The authors declare that they have no known competing financial interests or personal relationships that could have appeared to influence the work reported in this paper.

## Appendix Supplementary materials

renamed_f9dc5.docx Image, application 1

renamed_8baf4.docx Image, application 2

ANA Insertion.mp4 Image, video 3

ANA-Montage.mp4 Image, video 4

ANA-Vibration.mp4 Image, video 5

## Footnotes

Supplementary material associated with this article can be found in the online version at doi:10.1016/j.adro.2025.101896.

## References

[1] Zhang Y., Rumgay H., Li M., Cao S., Chen W. (2023). Nasopharyngeal cancer incidence and mortality in 185 countries in 2020 and the projected burden in 2040: Population-based global epidemiological profiling. JMIR Public Health Surveill.

[2] Chen Y.P., Chan A.T.C., Le Q.T., Blanchard P., Sun Y., carcinoma Ma J.Nasopharyngeal (2019). Lancet.

[3] Yu H., Yin X., Mao Y., Chen M., Tang Q., Yan S. (2022). The global burden of nasopharyngeal carcinoma from 2009 to 2019: An observational study based on the Global Burden of Disease Study 2019. Eur Arch Otorhinolaryngol.

[4] Yang G., Huang J., Sun J., Wang L. (2023). Elderly nasopharyngeal carcinoma patients (aged ≥70 years): Survival and treatment strategies. Cancer Med.

[5] Levendag P.C., Keskin-Cambay F., de Pan C. (2013). Local control in advanced cancer of the nasopharynx: Is a boost dose by endocavitary brachytherapy of prognostic significance?. Brachytherapy.

[6] Bacorro W.R., Agas R.A.F., Cabrera S.M.R. (2018). A novel applicator design for intracavitary brachytherapy of the nasopharynx: Simulated reconstruction, image-guided adaptive brachytherapy planning, and dosimetry. Brachytherapy.

[7] Holzapfel L. (2003). Nasal vs oral intubation. Minerva Anestesiol.

[8] Bhatia S.S., Kalra J.P.S., Chhabra RS. (2018). Nasopharyngeal airway dimensions in different dentofacial skeletal patterns. RUHS J Health Sci.

[9] Rohde R., Friedland DR. (2022). Clinical perspectives on nasopharyngeal morphology in humans. Anat Rec (Hoboken).

[10] Budynas R.G., Nisbett JK. (2011).

[11] Carey J., Emery D., McCracken P. (2006). Buckling test as a new approach to testing flexural rigidities of angiographic catheters. J Biomed Mater Res B Appl Biomater.

[12] Martino J., Harri K. (2019). Virtual shaker modeling and simulation, parameters estimation of a high damped electrodynamic shaker. Int J Mech Sci.

[13] Kumazaki Y., Hirai R., Igari M. (2020). Development of an HDR-BT QA tool for source position verification. J Appl Clin Med Phys.

[14] Strohmaier S., Zwierzchowski G. (2011). Comparison of (60) Co and (192) Ir sources in HDR brachytherapy. J Contemp Brachytherapy.

[15] International Atomic Energy Agency. Dosimetry in Brachytherapy – An International Code of Practice for Secondary Standards Dosimetry Laboratories and Hospitals, Technical Reports Series No. 492, IAEA, 2023.

[16] Pócza T., Zongor Z., Melles-Bencsik B., Tatai-Szabó D.Z., Major T., Pesznyák C. (2020). Comparison of three film analysis softwares using EBT2 and EBT3 films in radiotherapy. Radiol Oncol.

[17] Clijsters M., Khan M., Backaert W. (2024). Protocol for postmortem bedside endoscopic procedure to sample human respiratory and olfactory cleft mucosa, olfactory bulbs, and frontal lobe. STAR Protoc.

[18] Atar M., Kadayifci A. (2014). Transnasal endoscopy: Technical considerations, advantages and limitations. World J Gastrointest Endosc.

[19] Zhao A., Gao S., Greskovich J., Wilkinson DA. (2021). Pre-clinical dosimetry of a new six-channel applicator for high-dose-rate treatment of esophageal cancer. J Contemp Brachytherapy.

[20] Grassin-Delyle S., Buenestado A., Naline E. (2012). Intranasal drug delivery: An efficient and non-invasive route for systemic administration: Focus on opioids. Pharmacol Ther.

[21] Bassis C.M., Tang A.L., Young V.B., Pynnonen MA. (2014). The nasal cavity microbiota of healthy adults. Microbiome.

[22] Lemon K.P., Klepac-Ceraj V., Schiffer H.K., Brodie E.L., Lynch S.V., Kolter R. (2010). Comparative analyses of the bacterial microbiota of the human nostril and oropharynx. mBio.

[23] Jean-Joseph J., Balagiannis N., Scheppach M.W. (2025). Auxetic solution for enhanced esophageal brachytherapy applicator. J Contemp Brachytherapy.

[24] Maresova P., Hajek L., Krejcar O., Storek M., Kuca K. (2020). New regulations on medical devices in Europe: Are they an opportunity for growth?. Adm Sci.

[25] Hussien E., Hay D. (2022). Management of acute pain. Surgery (Oxford).

