# Supplementary material for: Development and Validation of the Augsburg Nasopharyngeal Applicator: Enhancing Efficacy in Nasal Route Brachytherapy
Source: Adv Radiat Oncol. 2025 Aug 30;10(11):101896. doi: 10.1016/j.adro.2025.101896 (PMC12509761; doi:10.1016/j.adro.2025.101896)
Supplement: renamed_f9dc5.docx [file mmc1.docx]

# Annex A – Mathematical Modeling of the Bending Mechanism

Precise control over the shape of the ANA catheter is crucial for accurate radiation delivery. This is achieved through an integrated bending mechanism involving a polyamide loop-shaped mandrel, a PVC corpus, and a nylon 6/6 catheter. The mandrel applies forces to the corpus, which transmits them to the catheter, resulting in a controlled bending action. This process is essential for shaping the applicator and preserving its mechanical integrity.

To mathematically model the bending behavior [1], we define the bending angle θ(s) as a function of arc length s along the catheter. Assuming planar bending, the position of any point on the catheter in Cartesian coordinates (x(s), y(s)) is given by:

x(s) = x₀ +$\int_{0}^{s} cos(\theta(\xi)) d\xi$
y(s) = y₀ + $\int_{0}^{s} sin(\theta(\xi)) d\xi$

where x₀ and y₀ are the initial coordinates.

The curvature κ(s) is the rate of change of the bending angle with respect to arc length:

κ(s) = dθ(s)/ds

The relationship between curvature and applied forces is governed by the Euler–Bernoulli beam equation:

EI ⋅ κ(s) = M(s)

where:
- E is the Young’s modulus of the catheter material,
- I is the second moment of area of the catheter cross-section,
- M(s) is the bending moment at position s.

The corpus modulates the mandrel’s forces before they reach the catheter. The bending moment at any point can be expressed as:

M(s) = F ⋅ d(s)

where F is the applied force and d(s) is the perpendicular distance from the force's line of action to the catheter’s neutral axis.

To account for both the catheter and corpus, we define an effective bending stiffness:

(EI)_effective = E_PVC I_PVC + E_catheter I_catheter

This allows the differential equation for bending to be written as:

(EI)_effective ⋅ κ(s) = F ⋅ d(s)

Solving this equation numerically yields the bending angle θ(s), which describes the shape of the catheter.

We also introduce a constraint to ensure that the curvature at any point does not exceed the critical value:

κ(s) ≤ κ_max

This constraint is necessary to prevent kinking, which can compromise performance by:
- Blocking the catheter lumen,
- Hindering manipulation and placement,
- Causing trauma during insertion or removal.

This model informs multiple aspects of ANA design [2]:
- Customization of flexibility for different anatomical configurations,
- Optimization of structure for uniform bending and accurate dose delivery,
- Estimation of insertion forces to minimize patient discomfort,
- Tuning of the adjustable perforation system for size adaptation,
- Material simulation to identify optimal mechanical properties.

**References**

1. [J. Tinsley Oden](https://onlinelibrary.wiley.com/authored-by/).An Introduction to Mathematical Modeling: A Course in Mechanics, 2011, ISBN:9781118105733. https://doi.org/10.1002/9781118105733

2. Callister, W. D., & Rethwisch, D. G. (2018). Materials Science and Engineering: An Introduction (10th ed.). Wiley. ISBN: 978-1-119-40549-8
