# Supplementary material for: Development and Validation of the Augsburg Nasopharyngeal Applicator: Enhancing Efficacy in Nasal Route Brachytherapy
Source: Adv Radiat Oncol. 2025 Aug 30;10(11):101896. doi: 10.1016/j.adro.2025.101896 (PMC12509761; doi:10.1016/j.adro.2025.101896)
Supplement: renamed_8baf4.docx [file mmc2.docx]

**Annex B – FEA of ANA**

We conducted a non-linear FEA using SOLIDWORKS (version 2023, Dassault

Systèmes, Waltham, MA, USA) to numerically solve the complex differential

equations governing catheter bending under large deformations [1]. This

approach discretizes the intricate catheter geometry into manageable elements,

enabling comprehensive analysis of its behavior under applied forces.

The non-linear FEA model is represented by the iterative system equation:

[K(θ)] {θ} = {F}

Where [K(θ)] is the non-linear stiffness matrix dependent on the current deformation

state, {θ} is the vector of nodal displacements (bending angles), and {F} is the vector

of applied forces. We solve this system iteratively using a Newton-Raphson method

, subject to the curvature constraint dθ/ds ≤ κmax, crucial for maintaining catheter

integrity.

Our model employs a fine mesh of beam elements (Fig. B1), with boundary conditions

accurately representing catheter-corpus interactions. The non-linear analysis

accounts for large deformations and potential material non-linearities, providing a

more accurate representation of the catheter's behavior under extreme bending

conditions [1, 2].

This FEA approach allows for precise modeling of the catheter's non-linear behavior,

considering material properties, applied forces, and bending constraints. It provides

critical insights into stress distributions, deformation patterns, and potential failure

modes under large deformations. By enabling virtual testing and iteration, this

method substantially reduces the time and cost associated with physical prototype

development and testing. This computational approach facilitates rapid design

improvements, streamlining the development process and optimizing resource

allocation [2].


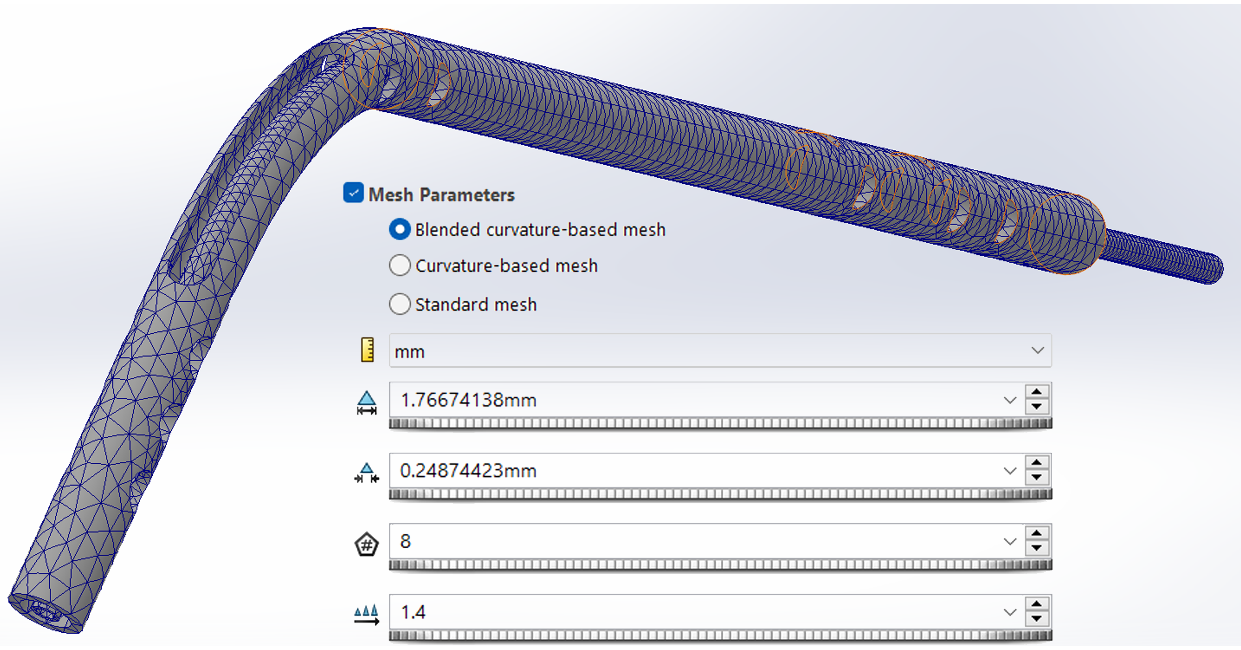


Figure B1. Mesh view of ANA used in the FEA.

**Model Creation and Material Assignment**

The nylon 6/6 catheter was meticulously modeled in Computer-Aided Engineering

(CAE) software to reflect its precise geometric dimensions The material properties

were assigned based on the characteristics of nylon 6/6, ensuring that the simulation

accurately represented the behavior of the actual material. The key material

properties used in the simulation include [2]:

Young's Modulus: 0.169 GPa

Poisson's Ratio: 0.35

Density: Around 1.2 g/cm³

In addition to the catheter, the assembly also included the corpus. The corpus was

modeled to fit seamlessly with the catheter, providing structural support while

allowing for the necessary movement and flexibility during use. The key material

properties for the PVC used in the simulation include [2]:

Young's Modulus: 0.003 GPa

Poisson's Ratio: 0.40

Density: Approximately 1.38 g/cm³

**Reference:**

1. Budynas RG, Nisbett JK. Shigley's Mechanical Engineering Design. 9th ed. New York: McGraw-Hill; 2011 ISBN: 0073529281.
2. Callister, W. D., & Rethwisch, D. G. (2018). Materials Science and Engineering: An Introduction (10th ed.). Wiley. ISBN: 978-1-119-40549-8
